# State of the art procedures towards reactive [^18^F]fluoride in PET tracer synthesis

**DOI:** 10.1186/s41181-023-00203-5

**Published:** 2023-10-12

**Authors:** Lizeth Y. F. Haveman, Danielle J. Vugts, Albert D. Windhorst

**Affiliations:** 1 Radiology and Nuclear Medicine, Amsterdam UMC, Vrije Universiteit Amsterdam, De Boelelaan 1117, Amsterdam, The Netherlands; 2 Chemistry and Pharmaceutical Sciences, Amsterdam Institute of Molecular and Life Sciences (AIMMS), Amsterdam, The Netherlands; 3 Cancer Center Amsterdam (CCA), Amsterdam, The Netherlands; 4 Neuroscience Amsterdam, Amsterdam, The Netherlands

**Keywords:** Radiopharmaceutical chemistry, Fluorine-18

## Abstract

**Background:**

Positron emission tomography (PET) is a powerful, non-invasive preclinical and clinical nuclear imaging technique used in disease diagnosis and therapy assessment. Fluorine-18 is the predominant radionuclide used for PET tracer synthesis. An impressive variety of new ‘late-stage’ radiolabeling methodologies for the preparation of ^18^F-labeled tracers has appeared in order to improve the efficiency of the labeling reaction.

**Main body:**

Despite these developments, one outstanding challenge into the early key steps of the process remains: the preparation of reactive [^18^F]fluoride from oxygen-18 enriched water ([^18^O]H_2_O). In the last decade, significant changes into the trapping, elution and drying stages have been introduced. This review provides an overview of the strategies and recent developments in the production of reactive [^18^F]fluoride and its use for radiolabeling.

**Conclusion:**

Improved, modified or even completely new fluorine-18 work-up procedures have been developed in the last decade with widespread use in base-sensitive nucleophilic ^18^F-fluorination reactions. The many promising developments may lead to a few standardized drying methodologies for the routine production of a broad scale of PET tracers.

## Background

Molecular imaging is the in vivo visualization of biological processes at the molecular level via the interaction of a probe with its target (Gu et al. 2011). Within the field of molecular imaging, positron emission tomography (PET) is a powerful, non-invasive preclinical and clinical nuclear imaging modality. The use of PET requires administration of a tracer radiolabeled with usually short-lived positron-emitting nuclides such as ^15^O, ^13^ N, ^11^ C, ^18^F, ^64^Cu, ^68^Ga or ^89^Zr. Although more positron emitting radionuclides find their way into PET applications, fluorine-18 remains the most frequently used radionuclide due to its favorable chemical and physical properties. A convenient half-life of 109.7 min, a clean decay profile (97% positron emission) and a low positron energy (maximum 0.635 MeV) resulting in high-resolution PET images (Van Der Born et al. 2017).

Several radiosynthetic methods are currently available for incorporation of fluorine-18 into biologically active compounds. In recent years, the unique and distinctive radiochemistry has been excellently reviewed (Van Der Born et al. 2017; Deng et al. 2019; Preshlock et al. 2016b). Although many different techniques have been implemented to improve the efficiency of the labeling reaction, only a few have dealt with the early key steps of the process, which consist of the removal of [^18^F]fluoride from its oxygen-18 enriched water ([^18^O]H_2_O) matrix and the generation of its reactive form. The landmark paper by Hamacher et al., describing a high yielding synthesis of 2-[^18^F]fluoro-2-deoxy-*D*-glucose ([^18^F]FDG), more or less defined the conventional route of producing reactive [^18^F]fluoride (Hamacher et al. 1986). This method starts with the production of fluorine-18 by proton irradiation of [^18^O]H_2_O in a cyclotron via the ^18^O(p,n)^18^F nuclear reaction. The radionuclide is obtained as strongly hydrated [^18^F]fluoride ions typically ranging from 50 to 200 GBq and with high molar activity (up to 1 TBq/µmol) (Aerts et al. 2010; Brichard and Aigbirhio 2014; Pees et al. 2018; Wessmann et al. 2012). The [^18^F]fluoride is trapped on an anion-exchange resin, typically a Waters Sep-Pak QMA, allowing the removal and recovery of expensive target [^18^O]H_2_O. The radioactivity is then eluted with a ~ 10% aqueous acetonitrile (MeCN) solution containing an inorganic base and cryptand, typically K_2_CO_3_/K_222_ (20 : 40 µmol). Subsequent drying of the eluate by repeated azeotropic distillation with MeCN provides the [^18^F]fluoride starting material. Finally, the residual anhydrous [^18^F]KF/K_222_ complex is taken up in a polar aprotic solvent suitable for the subsequent labeling reaction.

**Fig. 1:**
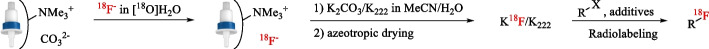
Overview of the conventional route for the purification and drying of aqueous [^18^F]fluoride with an anion-exchange cartridge

Although optimized, the overall procedure is time-consuming in the context of ^18^F-radiochemistry (around 10–15 min) and can lead to losses of significant amounts of radioactivity (up to 30%) due to unspecific adsorption on the reactor surface during azeotropic drying (Brichard and Aigbirhio 2014; Pees et al. 2018; Wessmann et al. 2012). This procedure is also complex to automate and miniaturize, e.g. for the use in microfluidic devices, and it is hard to control the precise hydration state of fluoride leading to irreproducible results. Most importantly, substantial amounts of base are needed to isolate [^18^F]fluoride from the cartridge thereby limiting its synthetic utility in the subsequent radiofluorination. The principles of the conventional work-up still remain in modern radiofluorination methods, but significant changes into especially the elution step, but also the trapping and drying stages have been introduced in the last decade (Fig. 1). In this review, we provide an overview of the strategies and recent developments in the production of reactive [^18^F]fluoride and their applications in PET tracer synthesis in the last decade. To demonstrate that these isolation and activation protocols are indeed valuable assets, the technical ease, efficiency (radiochemical yield (RCY), activity yield (AY), radiochemical conversion (RCC), molar activity (A_m_) and time will be discussed in each case (Coenen et al. 2017; Herth et al. 2021). Where applicable, the proof-of-concept application and routine production of representative and clinically relevant ^18^F-labelled radiopharmaceuticals under conditions of good manufacturing practice (GMP) are described.

## Main text

### Trapping on anion-exchange cartridges

During trapping, the negatively-charged [^18^F]fluoride ions are attracted to a positively-charged resin and exchange with the negatively charged ions to adsorb to the surface while the [^18^O]water continues to the outlet. Depending on the functional groups bound to the resin, the cartridges can be strongly or weakly basic. In addition, the negatively charged ions used in the preconditioning step are a major contributor to the basicity of the reaction mixture after elution. Hence, the type and size of anion-exchange resin as well as the preconditioning agent can substantially influence the work-up procedure of fluorine-18 with regard to azeotropic drying, losses of significant amounts of [^18^F]F^−^ on the reactor surfaces or the amount of base and phase transfer catalyst needed to elute [^18^F]F^−^.

### Solid support of the anion-exchange cartridge

Generally, [^18^F]F^−^ is extracted from the [^18^O]water on a polymer resin impregnated with quaternary methylammonium salts (QMA). Due to their strong anion exchange capacity, several weaker quaternary and tertiary ammonium salts have been developed to reduce the amount of base and cryptand required to elute the [^18^F]F^−^ and to perform on-resin radiolabeling reactions. Alternatively, main group elements, such as boranes and phosphazenes, have been studied in the design of cartridge resins for the preparation of dry [^18^F]fluoride in the absence of an azeotropic drying step.

#### Quaternary ammonium salts

**Fig. 2:**
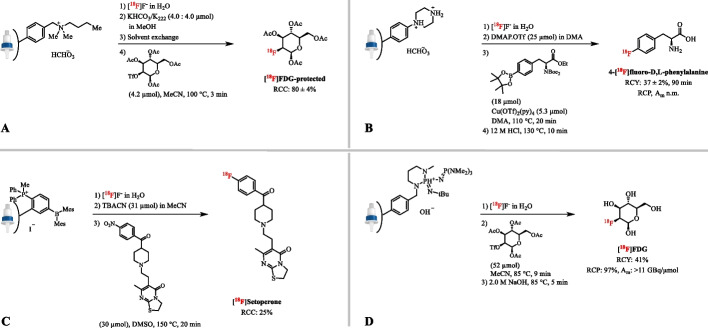
** A** protected [^18^F]FDG with a mixed-mode quaternary ammonium salt resin (MAX, Oasis). **B** Radiosynthesis of 4-[^18^F]FPhe from with a tertiary ammonium based anion-exchange resin (Oasis WAX). **C** Radiosynthesis of [^18^F]Setoperone with a phosphonium borane [(Ph_2_MeP)C_6_H_4_(BMes_2_)]^+^ anion-exchange resin. **D** Radiosynthesis of [^18^F]FDG with a phosphazene base (P_2_tBu) supported anion-exchange resin; n.m.: not mentioned

Resins based on quaternary ammonium salts were investigated with a custom-synthesized 4-(*N,N*-dialkylamino)-pyridinium functionalized anion-exchange resin for the collection, drying and on-column ^18^F-radiolabeling reaction (Mulholland et al. 1989; Toorongian et al. 1990). Problems such as solubility of counter anions and removal of phase transfer catalyst reagents were eliminated, resulting in the radiosynthesis of [^18^F]FDG with an average AY of 41 ± 15.6% (*n =* 104), which is comparable to the classical method (44 ± 4% AY, *n* = 7). In addition, Aerts et al. described the use of long alkyl chain quaternary ammonium salts as resin to quantitatively trap [^18^F]fluoride and recover > 85% of the trapped activity with 1 mL of a non-protic solvent (Aerts et al. 2010). However, the co-eluted concentration of water is higher than that obtained after classical azeotropic drying of a K_2_CO_3_/K_222_ solution, which might cause problems in water-sensitive reactions such as Cu-catalyzed radiofluorinations. Furthermore, the quantitative recovery of [^18^F]F^−^ (98.5 ± 1.0%, *n* = 23) with just 2 µmol of K_222_/KHCO_3_ in MeOH has been described using a commercially available, mixed-mode quaternary ammonium salt resin (MAX, Oasis) (Fig. 2A) (Iwata et al. 2017). The amount of K_2_CO_3_/K_222_ could be even further reduced by a subsequent cation-exchange cartridge (Oasis MCX) (Iwata et al. 2018). This procedure enabled the microscale radiosynthesis several clinically relevant precursors in RCC’s comparable to those reported in the literature. Importantly, since the solvent scale is small, poor reproducibility of the radiosyntheses was obtained.

#### Tertiary ammonium salts

Instead of quaternary amines, a commercially available piperazine based anion-exchange resin (Oasis WAX) has been studied for trapping of the aqueous [^18^F]F^−^ and its release by elution with a weak anion-exchanger, in this case pyridinium sulfonate (25 µmol) in DMA (78% elution efficiency) (Fig. 2B) (Antuganov et al. 2019). The reactive [^18^F]fluoride was then directly used in a Cu-catalyzed radiofluorination reaction obtaining high RCCs for a series of simple arylBPin substrates and a RCY of 35–38% (*n* = 2) for 4-[^18^F]FPhe within 90 min.

#### Phosphonium borane salts

[^18^F]Fluoride has been prepared according to a simple two-step sequence including trapping of aqueous [^18^F]F^−^ on a cartridge pre-loaded with the phosphonium borane [(Ph_2_MeP)C_6_H_4_(BMes_2_)]^+^, followed by quantitative release of [^18^F]F^−^ from the cartridge using a solution of tetrabutylammonium cyanide (TBACN) (one equivalent with respect to borane) in anhydrous MeCN (Fig. 2C) (Perrio et al. 2017). Loading of the cartridge with borane salt and using equimolar amounts of TBACN to replace [^18^F]F^−^ on the cartridge as well as to convert the excess borane salt to borane-CN are a prerequisite. Subsequent radiofluorination was successfully applied to model compounds and to the synthesis of [^18^F]setoperone (25% RCC) (Crouzel et al. 1988).

#### Phosphazene bases

Based on the work by Lemaire et al. on the radiofluorination with phosphazene bases, polymer supported phosphazene bases (P_2_tBu and P_2_PEG) have been used for the efficient extraction of fluorine-18 from [^18^O]H_2_O. Treatment of the resin with [^18^O]H_2_O forms the P_2_tBuH^+^OH^−^ ion-pair and [^18^F]F^−^ is trapped via the [^18^F]F^−^/OH^−^ anion-exchange to form the P_2_tBuH^+^[^18^F]F^−^ adduct (Fig. 2D) (Lemaire et al. 2010; Mathiessen and Zhuravlev 2013). The resin trapped > 97% of [^18^F]F^−^ after which the solution of substrate was passed through the column effecting on-column radiofluorination for several model compounds. An advantage of this method compared to the ones mentioned above, is that the column can be reused without any loss in [^18^F]F^−^ trapping efficiency. However, repeated use is limited to 3 runs. [^18^F]FDG was produced with a RCY of 41% (*n* = 1) on a 120 GBq scale with an A_m_ >11 GBq/µmol, which is comparable to the benchmark production of [^18^F]FDG (44 ± 4% AY, *n* = 7).

### Size of the anion-exchange cartridge

Reducing the size of the anion-exchange cartridge has also been considered as an alternative for reducing water and/or base content. In macro-scale reactions, this method has only been applied once in the radiosynthesis of [^18^F]fluoroazomycin arabinosid ([^18^F]FAZA) (Hayashi et al. 2011). A small home-made QMA cartridge containing 25 mg of the QMA resin, compared to the conventional 130 mg, resulted in a high RCY of 52.4 ± 5.3% (*n* = 8) with just 5.0/6.0 µmol of K_2_CO_3_/K_222_ within 50.5 ± 1.5 min.

#### Miniature anion-exchange cartridges

One of the first reports demonstrated concentration of [^18^F]fluoride (up to 10 GBq) in 1–5 mL of [^18^O]H_2_O with a custom-build miniature cartridge (~ 5 µL bed volume) packed with QMA resin (Bejot et al. 2011). Trapping and release of [^18^F]F^−^ with only 2.1 µmol K_2_CO_3_ in H_2_O followed by 4.8 µmol K_222_ in MeCN resulted in 95–97% recovery of [^18^F]F^−^ with a total processing time of ~ 11 min. Since then, commercial micro-cartridges (OptiLynx, 5 µL bed volume) have enabled the concentration of [^18^F]fluoride (up to 110 GBq) to a final volume of 5–45 µL (Chao et al. 2018; Elizarov et al. 2010; Lebedev et al. 2013). On average, > 99% of [^18^F]F^−^ is trapped and > 92% is subsequently released with a typical K_2_CO_3_/K_222_ mixture (0.50/0.60 µmol) in ~ 3 min. Noteworthy, the OptiLynx cartridge filled with Chromabond PS-HCO_3_ resin has been successfully used in the preparation of human doses of 5-[^18^F]fluorouracil (Hoover et al. 2016). Efficient elution of [^18^F]F^−^ (81%) was obtained with a MeCN/H_2_O mixture of K_3_PO_4_/18-crown-6 (0.5/3.0 µmol). However, a major drawback of these procedures is the inconvenient manual packing of resin in the cartridge and the lack of reproducibility that this operation implies.

#### Miniature anion-exchange particles

For the integration of anion-exchange resins on the microfluidic device, several techniques have been reported. First, solution suspended anion-exchange beads have been reported by Lee et al. (2005). After trapping of fluorine-18 on a chip (26 MBq in 1 µL [^18^O]H_2_O), 18 MBq was recovered by elution with 5 nmol of aqueous K_2_CO_3_ solution and an azeotropic drying step (69% efficiency). The method provided 4 MBq of [^18^F]FDG with a RCY of 38% within 14 min. In addition, Leonardis et al. manually loaded recyclable anion-exchange resin particles (Chromabond PS-HCO_3_) into on-chip cavities (Leonardis et al. 2011). The trapping efficiency of 5–7 GBq [^18^F]fluoride in 4 mL [^18^O]H_2_O was > 90% and ≥ 95% of the trapped radioactivity was eluted with 9 µmol K_2_CO_3_ in 250 µL MeCN/H_2_O within 6 min. Furthermore, concentration of [^18^F]fluoride has been demonstrated with 10–15 mg embedded QMA resin (Sep-Pak Accell Plus cartridge) (Salvador et al. 2017). For activities of 19 GBq, trapping of 2 mL of [^18^F]fluoride in [^18^O]H_2_O was achieved with 98% efficiency. The trapped activity could then be eluted into a volume of 20 µL K_2_CO_3_ (7.2 µmol) with > 87% recovery. Instead of filling on-chip cavities, an insert element pre-filled with QMA resin (Sep-Pak® Light QMA) has been integrated on-chip (Rensch et al. 2014). 243–394 MBq in 100–500 µL [^18^O]H_2_O was concentrated with ~ 90% efficiency, which is comparable to conventional cartridge performance.

#### Anion-exchange monolith

Integration of the cartridges or resin particles in the chip is typically a challenge. Therefore Ismail et al. used a functionalized polymer monolith (polystyrene imidazolium chloride) instead of packed resin particles (Ismail et al. 2014). Trapping of [^18^F]fluoride solutions (1.5–7.4 MBq) had an efficiency of 97 ± 4% (*n* = 39) and it was shown theoretically that higher activities could be trapped by extending the length of the monolith. Various eluents were tested, recovering at least > 85% of the activity in a volume of 200 µL.

### Preconditioning of the anion-exchange cartridge

Preconditioning of the QMA cartridge has been shown to have a significant effect on the [^18^F]F^−^ recovery in certain cases. For example, when using an eluent of iodonium salt precursor in MeOH, the presence of HCO_3_^−^ anions on the anion-exchange resin are key. The rapid and efficient exchange of the diaryl iodonium salts counteranions with hydrogen carbonate results in an elution efficiency of > 90%. In contrast, other resin anions such as Cl^−^, BF_4_^−^, CF_3_SO_3_^−^ and CF_3_CO_2_^−^ lowered the elution yields to 14–49% (Maisonial-besset et al. 2018; Pauton et al. 2019).

#### Oxalate and triflate counteranions (C_2_O_4_^2−^, OTf^−^)

**Fig. 3:**
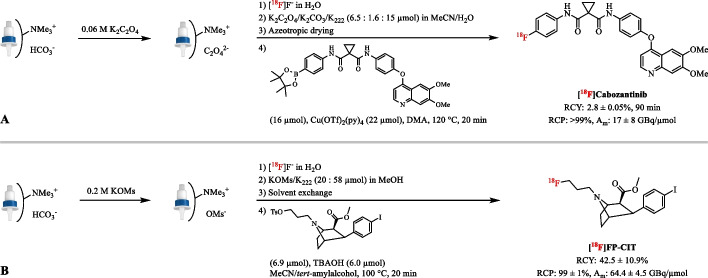
Radiosynthesis of [^18^F]Cabozantinib and [^18^F]FP-CIT after preconditioning the anion-exchange resin with an aqueous potassium oxalate (**A**) and potassium mesylate (**B**) solution, respectively

For Cu-mediated ^18^F-radiolabeling reactions, it has been reported that a combination of appropriate preconditioning and concentration of base in the eluent can improve [^18^F]F^−^ recovery when eluting with weak bases (pK_a_ < 7) as illustrated by [^18^F]Cabozantinib (Lien et al. 2018; Mossine et al. 2017). In a full-scale experiment using 15 GBq up to 40% of [^18^F]F^−^ was lost due to sticking to the reaction vessel or on the anion-exchange column resulting in only 0.7% RCY. Modifying the preconditioning (oxalate solution) and the elution (1.6/6.5/15 µmol of K_2_CO_3_/K_2_C_2_O_4_/K_222_) resulted in only 13% loss of activity, with the RCY increasing to 2.8 ± 0.05% (*n* = 4) and A_m_ of 17 ± 8 GBq/µmol (Fig. 3A). In general, when using weakly basic eluents such as K_2_CO_3_/K_2_C_2_O_4_/K_222_ and K_2_CO_3_/KOTf, the anion-exchange cartridge should be preconditioned with the corresponding weak counteranions, i.e. oxalate and triflate, respectively (Cai et al. 2020; Guibbal et al. 2019; Li et al. 2019a; McCammant et al. 2017; Preshlock et al. 2016a; Wu et al. 2019). Also when using copper complexes as elution agents, preconditioning with aqueous LiOTf solution can result in higher [^18^F]F^−^ recovery, but strongly depends on the type of QMA cartridge used (Lahdenpohja et al. 2019).

#### Mesylate and phosphate counteranions (OMs^−^, HPO_4_^−^, PO_4_^2−^)

In aliphatic radiofluorination reactions, controlling the base amount has been achieved by changing the bicarbonate counteranions on the resin to the inert mesylate anion, using 0.2 M of KOMs solution in the preconditioning step (Lee et al. 2011b, 2012). [^18^F]F^−^ can then be eluted with only 20 µmol KOMs resulting an eluate without any basic components. The exact amount of base or wanted precursor/base ratio can then be directly added to the reaction vessel. For example, ^18^F-incorporation yields for 3′-deoxy-3′-[^18^F]fluorothymidine ([^18^F]FLT) synthesis were 46.3 ± 5.5% (*n* = 3) and 17.79% (*n* = 1) for KOMs and K_2_CO_3_ preconditioned resins, respectively (Lee et al. 2012). Remarkably, in the synthesis of *N*-[^18^F]fluoropropylcarbomethoxyiodophenyl-nortropane ([^18^F]FP-CIT) the amount of base during nucleophilic ^18^F-fluorination was finely controlled by adding 6 µmol tetrabutylammonium hydroxide (TBAOH) to the reactor directly (Lee et al. 2011b). Over 192 preparations of [^18^F]FP-CIT have been performed with a mean RCY of 42.5 ± 10.9% and A_m_ of 64.4 ± 4.5 GBq/µmol (Fig. 3B). Furthermore, di- and tribasic phosphates have also been reported as counteranions in aliphatic fluorination reactions, but application to a pharmaceutically relevant compound has not been reported (Seo et al. 2011).

### Elution of anion-exchange cartridges with non-conventional bases, cryptands, solvents and additives

After trapping, the [^18^F]fluoride is eluted from the cartridge. Over the years several methods have been examined to optimize, but especially simplify this process. Multiple mixtures of anions and cryptands of both high and low basicity have successfully been applied in the elution of [^18^F]F^−^ from an anion-exchange cartridge. In addition, solvents ranging from aqueous polar aprotic to polar protic and even charged ones have been used to significantly reduce the time of the subsequent azeotropic drying step. Moreover, instead of bases and cryptands, the elution has been performed with precursors serving as the cation. Finally, ways of making the radiolabeling reactions tolerant to water have been given attention in order to omit a drying step.

### Elution with weakly basic potassium salts and cryptand in MeCN/H_2_O

Typically [^18^F]fluoride is extracted from QMA resins by elution with a ~ 10% aqueous MeCN solution containing relatively large amounts of potassium salts and cryptand, e.g. K_2_CO_3_/K_222_ (20:40 µmol). However, also weak bases and neutral phase transfer catalysts can be used for this purpose. So far, these eluents have been tailored to meet the specific needs of a subsequent reaction of interest.

#### Potassium carbonate/Kryptofix-222 (K_2_CO_3_/K_222_)

A very straightforward method for decreasing the amount of K_2_CO_3_/K_222_ is to omit the trapping/elution step of [^18^F]F^−^ and instead to directly evaporate the target enriched water. In the preparation of (*S*)-*N*-[(1-allyl-2-pyrrolidinyl)methyl]-5-(3-[^18^F]fluoropropyl)-2,3-dimethoxybenzamide ([^18^F]Fallypride) the amount of K_2_CO_3_/K_222_ was only 2.5–5 µmol by evaporating the [^18^O]H_2_O together with K_222_ and K_2_CO_3_ (Mukherjee et al. 1995). However, evaporation of a large volume of [^18^O]H_2_O is time-consuming and expensive. As an alternative, decreasing the amount of K_2_CO_3_ but maintaining the amount of K_222_ compared to the classical conditions, sometimes combined with using a larger amount of water, has proven to be successful in the radiolabeling of 2-nitropurine-based nucleosides, e.g. 2-[^18^F]Fludarabine (Guillouet et al. 2014; Marchand et al. 2010, 2016). Similarly, the high-base labeling conditions can be adapted by eluting [^18^F]F^−^ with a small aliquot of the conventional K_2_CO_3_/K_222_ solution (Zlatopolskiy et al. 2015). For example, a solution of K_2_CO_3_ (0.87 µmol) and K_222_ (1.5 µmol) led to efficient elution of [^18^F]F^−^ from the QMA cartridge (82 ± 2%, n = 27), which resulted in the production of [^18^F]FAMTO in a RCY of 18 ± 2% with an A_m_ of 105 ± 39 GBq/µmol from EOB within 120 min (Fig. 5A) (Bongarzone et al. 2019). In contrast, the formation of [^18^F]FAMTO under the classical conditions (K_2_CO_3_/K_222_ 12/20 µmol) was not observed.

#### Potassium carbonate/potassium oxalate/Kryptofix-222 (K_2_CO_3_/K_2_C_2_O_4_/K_222_)

**Fig. 4:**
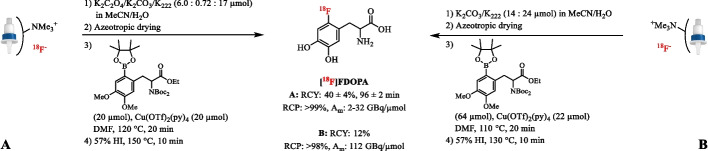
Radiosynthesis of [^18^F]FDOPA after elution of [^18^F]fluoride with a moderately basic K_2_CO_3_/K_2_C_2_O_4_/K_222_ eluent (**A**) and the conventional K_2_CO_3_/K_222_ solution (**B**)

Soon after the landmark paper by Hamacher et al., a system with potassium oxalate (K_2_C_2_O_4_) as base and minimal amounts of K_2_CO_3_ (≤ 0.36 µmol) was reported for the radiofluorination of butyrophenone neuroleptics (Hamacher and Hamkens 1995; Katsifis et al. 1993). The conventional strong basic K_2_CO_3_/K_222_ system led to degradation of the tosylated precursors, but with the alternative system ^18^F-incorporation was facilitated. This moderately basic eluent has been used in the nucleophilic substitution of several aromatic scaffolds including those of clinically relevant radiotracers such as [^18^F]-3-fluoro-5-[(pyridin-3-yl)ethynyl]benzonitrile ([^18^F]FPEB), [^18^F]Flumazenil ([^18^F]FMZ), *meta*-[^18^F]fluorobenzylguanidine ([^18^F]mFBG) and *L*-3-[^18^F]fluoro-α-methyltyrosine ([^18^F]FMT) (Hamill et al. 2005, 2011; Hostetler et al. 2011a, b; Labas et al. 2011). Following the K_2_C_2_O_4_/K_2_CO_3_ elution protocol, Preshlock et al. reported the successful Cu-mediated nucleophilic ^18^F-fluorination of clinical PET tracers from their arylboronic ester precursors. Using this protocol, degradation of the base-sensitive copper catalyst was prevented resulting in isolated RCY’s up to 39% and A_m_ >100 GBq/µmol (Preshlock et al. 2016a). Exemplary is the clinical production of 6-[^18^F]fluoro-*L*-dihydroxy-phenylalanine ([^18^F]FDOPA). By using K_2_CO_3_/K_2_C_2_O_4_/K_222_ (0.72/6.0/17 µmol), a dose of 2.18 GBq of [^18^F]FDOPA could be isolated corresponding to 40 ± 4% RCY (*n* = 5), while classical elution of the QMA cartridge with K_2_CO_3_ (14 µmol) and K_222_ (24 µmol) gave access to a clinical dose of [^18^F]FDOPA in 12% RCY (*n* = 1) (Fig. 4) (Tredwell et al. 2014). Since then, several examples of the radiofluorination of aryl boronic ester substrates with K_2_CO_3_/K_2_C_2_O_4_/K_222_ as eluent for the QMA cartridge have been reported (Ahmed et al. 2019; Bratteby et al. 2020; Clemente et al. 2019; Guibbal et al. 2020; Haider et al. 2019; Prause et al. 2018). Besides preventing degradation of base-sensitive precursors and catalysts, K_2_C_2_O_4_ has proven to be of aid in reducing racemization in substrates with a chiral center. The initial radiosynthesis of *L*-*S*-(3-[^18^F]fluoropropyl)homocysteine ([^18^F]-*L*-FPHCys) under conventional radiolabeling conditions resulted in a racemization of 25% towards [^18^F]-*D*-FPHCys due to proton abstraction of the *R*-hydrogen (Bourdier et al. 2011b). Under identical reaction conditions except for the use of non-nucleophilic K_2_CO_3_/K_2_CO_4_ (0.36/16 µmol) instead of K_2_CO_3_ (14 µmol), [^18^F]-*L*-FPHCys was obtained from the tosylate precursor without racemization in a AY of 20 ± 5% and radiochemical and enantiomeric purity of > 98% in 65 min.

#### Potassium carbonate/potassium triflate (K_2_CO_3_/KOTf)

To limit the quantities of strongly basic K_2_CO_3_ in Cu-mediated radiofluorination reactions of boron precursors, Mossine et al. examined the elution of [^18^F]F^−^ with a weakly basic combination of KOTf and K_2_CO_3_ (27/0.36 µmol) (Mossine et al. 2015). With this new eluent, 80% of the [^18^F]F^−^ was recovered from the QMA and no loss of radioactivity during the azeotropic drying step was observed. Noteworthy, these weakly basic [^18^F]F^−^ elution conditions have been used in the preparation of PET tracers that could not be obtained with conventional methods on a preparative scale from both boron and stannane precursors (Elie et al. 2019; Makaravage et al. 2016; Wu et al. 2019). For example, radiotracers for the in vivo imaging of TrkA/B/C receptors and oncogenic Trk fusion proteins 2019; Li et al. 2019a).

#### Potassium dihydrogen phosphate/Kryptofix-222 (KH_2_PO_4_/K_222_)

**Fig. 5:**
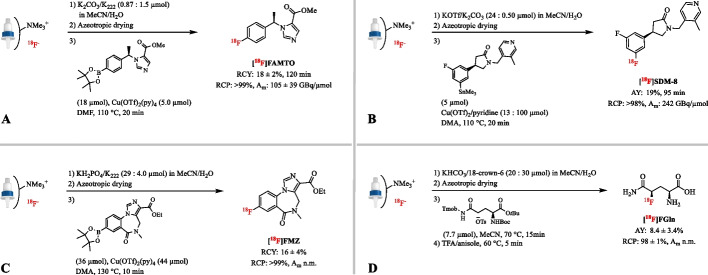
** A** Radiosynthesis of [^18^F]FAMTO after elution of [^18^F]fluoride with an aliquot of the conventional K_2_CO_3_/K_222_ solution. **B** Radiosynthesis of [^18^F]SDM-8 after elution of [^18^F]fluoride with the weakly basic K_2_CO_3_/KOTf eluent. **C** Radiosynthesis of [^18^F]FMZ after elution of [^18^F]fluoride with the moderately basic KH_2_PO_4_/K_222_ eluent. **D** Radiosynthesis of [^18^F]FGln after elution of [^18^F]fluoride with the moderately basic KHCO_3_/18-Crown-6 eluent; n.m.: not mentioned

Although not common, a solution of KH_2_PO_4_ (29 µmol) with K_222_ (40 µmol) has been used for elution of [^18^F]F^−^ in the automated synthesis of [^18^F]FMZ from its boronic ester precursor (Fig. 5C) (Preshlock et al. 2016a). [^18^F]FMZ was isolated in 16 ± 4% RCY (*n* = 10) comparable to the 19% RCY (*n* = 1) obtained by using K_2_C_2_O_4_/K_2_CO_3_ (0.72/6.0 µmol) and K_222_ (17 µmol) as eluent.

#### Potassium bicarbonate/18-crown-6 (KHCO_3_/18-Crown-6)

In specific cases, not only changing to a mild basic agent is necessary, but also to a neutral phase transfer catalyst. In the radiolabeling reaction of 4-[^18^F]fluoroglutamine all four possible isomers and a significant amount of elimination byproducts were formed in the presence of K_222_ and K_2_CO_3_ (Qu et al. 2011). The less basic KHCO_3_/18-Crown-6 (13/28 µmol) catalyst system led to the successful automated preparation of the desired *L*-glutamine isomer (2* S*,4*R*)-4-[^18^F]fluoroglutamine (4-[^18^F]FGln) in 8.4 ± 3.4% AY (n = 10) and high stereoisomeric purity (> 91 ± 8%) (Fig. 5D) (Lieberman et al. 2011).

### Elution with tetraalkylammonium salts as base and cryptand in MeCN/H_2_O

Tetraalkylammonium salts have an enhanced solubility in organic solvents and are therefore widely used as an alternative to the K_2_CO_3_/K_222_ system. Generally, an ion exchange between the [^18^F]F^−^ and the counter-anion of the ammonium salt occurs during elution of the activity from the QMA resin with a tetraalkylammonium salts solution. The tetraalkylammonium/[^18^F]F^−^ complex serves then as the phase transfer catalyst. The different alternatives will hereafter be discussed.

#### Tetrabutylammonium hydroxide (TBAOH)

Early on, Parent et al. reported the labeling of *N*-(3-fluoro-4-nitronaphthyl)-*cis*-5-norbornene-endo-2,3-dicarboxylic imide (3-[^18^F]F-NNDI) through an extremely mild, S_N_Ar displacement reaction of an *o*-nitro-activated arene trimethylammonium salt with fluorine-18 by using TBAOH (2 µL) (Parent et al. 2006). This procedure allowed the preparation of 3-[^18^F]F-NNDI in 70–81% RCY.

#### Tetrabutylammonium bicarbonate (TBAHCO_3_)

**Fig. 6:**
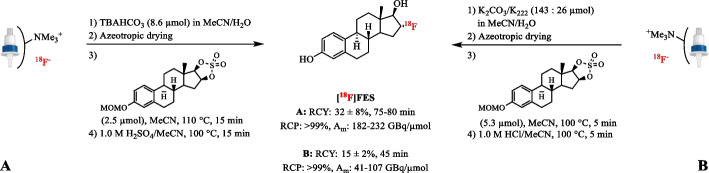
Radiosynthesis of [^18^F]FES after elution of [^18^F]fluoride with a TBAHCO_3_ solution (**A**) and the conventional K_2_CO_3_/K_222_ solution (**B**)

**Fig. 7:**
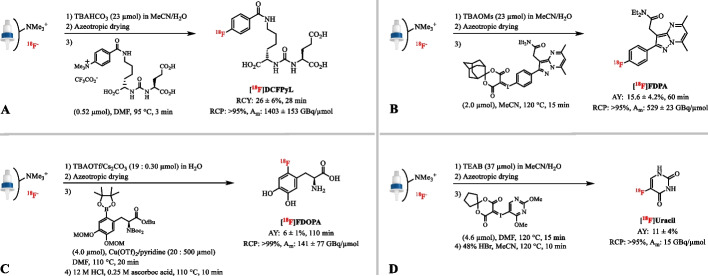
** A** Radiosynthesis of [^18^F]DCFPyL after elution of [^18^F]fluoride with a TBAHCO_3_ solution. **B** Radiosynthesis of [^18^F]FDPA after elution of [^18^F]fluoride with a very mild basic TBAOMs solution. **C** Radiosynthesis of [^18^F]FDOPA after elution of [^18^F]fluoride with a weakly basic TBAOTf solution. **D** Radiosynthesis of [^18^F]Uracil after elution of [^18^F]fluoride with a TEAB solution

Currently, TBAHCO_3_ is the most commonly used ammonium salt for the preparation of [^18^F]TBAF. It has proven its value in both base-sensitive aliphatic and aromatic nucleophilic substitution reactions. For example, TBAHCO_3_ (8.6 µmol) increased the RCY of 16α-[^18^F]fluoroestradiol ([^18^F]FES) from the cyclic sulfonate precursor up to 32 ± 8% compared to a RCY of 15 ± 2% with K_2_CO_3_/K_222_ (13/26 µmol) as phase transfer catalyst (Fig. 6) (Fedorova et al. 2018; Knott et al. 2011; Wang et al. 2020b). Furthermore, Chang et al. showed that the synthesis of 3-[^18^F]fluoro-1-(2′-nitro-1′-imidazolyl)-2-propanol ([^18^F]FMISO) using [^18^F]TBAF instead of [^18^F]KF/K_222_ in the substitution of the tosyl moiety increased the RCY from 15 ± 3% to 30 ± 5% (AY, *n* = 75) (Chang et al. 2007; Cherif et al. 1994). In addition, TBAHCO_3_ (30 µmol) provided equally good RCYs as the classical K_2_CO_3_/K_222_ system (51/58 µmol) (48 ± 3% and 54.4 ± 2.9% RCY (*n* = 3), respectively) in the [^18^F]FMISO synthesis with the THHP protecting group (Nandy et al. 2010; Oh et al. 2005). In the case of small peptidic radiotracers, the use of [^18^F]TBAF results in clean reaction profiles with high yields, while classical bases such as K_2_CO_3_ and KOAc typically result in lower yields. Interestingly, Dornan et al. reported the efficient production of 2-(3‐{1‐carboxy‐5‐[(6‐[^18^F]fluoro‐pyridine‐3‐carbonyl)‐amino]‐pentyl}‐ureido)‐pentanedioic acid ([^18^F]DCFPyL) using a direct, single‐step radiofluorination reaction without the use of protecting groups in the presence of [^18^F]TBAF (Fig. 7A) (Dornan et al. 2018). This simplified approach enabled automated preparation of [^18^F]DCFPyL in 26 ± 6% AY (n = 7) and with high A_m_ (1403 ± 153 GBq/µmol) within 28 min. Presently, [^18^F]TBAF has allowed the preparation of clinically-relevant radiotracers, via fully-automated procedures including *O*-(2-[^18^F]fluoroethyl)-*L-*tyrosine ([^18^F]FET), 16β-[^18^F]fluoro-5α-dihydrotestosteron ([^18^F]FDHT), 2′-deoxy-2′-[^18^F]fluoro-5-methyl-1-β-*D*-arabinofuranosyluracil ([^18^F]FMAU), prostate‐specific membrane antigen ([^18^F]PSMA-1007) and [^18^F]DCFPyl (Bouvet et al. 2016; Cardinale et al. 2017; Lakshminarayanan et al. 2017; Lazari et al. 2015; Li et al. 2011; Ravert et al. 2016; Shamni et al. 2019).

#### Tetrabutylammonium methanesulfonate (TBAOMs)

The use of TBAOMs (36–40 µmol) as very mild base for the generation of [^18^F]TBAF has significantly increased in recent years due to the development of iodonium containing precursors i.e. diaryliodonium salts and spirocyclic iodonium ylides (SCIDY) (Vavere et al. 2018). These precursors are generally unstable under basic conditions and undergo radical-induced decomposition under the conventional radiolabeling conditions with [^18^F]KF/K_222_ (Lee et al. 2011a; Shi et al. 2016). For example, in the radiolabeling of the TSPO PET ligand *N,N*-diethyl-2-(2-(4-[^18^F]fluorophenyl)-5,7-dimethylpyrazolo[1,5- *a*]pyrimidine-3-yl)-acetamide ([^18^F]FDPA) only trace amounts of product were detected in the presence of K_2_CO_3_/K_222_. In contrast, TBAOMs (36 µmol) as eluent provided > 95% [^18^F]F^−^ elution efficiency and 4.2% AY (*n* = 3) and A_m_ of 529 ± 23 GBq/µmol within 60 min (Fig. 7B) (Wang et al. 2017, 2020a). TBAOMs as phase transfer catalyst has enabled the efficient and automated production of several PET radiotracers from their SCIDY precursors, e.g. *N*-(2,5-dimethoxybenzyl)-*N*-(5-[^18^F]fluoro-2- phenoxyphenyl)acetamide ([^18^F]DAA1106) (Fujinaga et al. 2018; Kumata et al. 2018).

#### Tetrabutylammonium triflate (TBAOTf)

The [^18^F]KF generated from the weakly basic K_2_CO_3_/KOTf eluent causes side reactions e.g. protodeborylation and/or hydroxydeborylation in Cu- mediated fluorination reactions of arylboronic pinacol esters due to poor dissolution (Mossine et al. 2018). To facilitate efficient elution from QMA resins and rapid dissolution of [^18^F]F^−^, the weak nitrogenous base TBAOTf (13–19 µmol) has been used (Zhang et al. 2017a, b). Noteworthy, the only validated production for human use of [^18^F]FDOPA from [^18^F]F^−^ uses an aqueous eluent consisting of TBAOTf and Cs_2_CO_3_ (19/0.30 µmol) as a replacement for KOTf and K_2_CO_3_, respectively (Fig. 7C). Although, the AY and A_m_ (6 ± 1% and 141 ± 77 GBq/µmol) were comparable to the RCY and A_m_ obtained under conventional conditions (12% and 112 GBq/µmol), the chemical purity of [^18^F]FDOPA was improved, qualifying it for clinical use (Mossine et al. 2019).

#### Tetraethylammonium bicarbonate (TEAB)

As [^18^F]TBAF, [^18^F]TEAF has been discovered as an alternative to the [^18^F]KF/K_222_ system in the radiolabeling of diaryliodonium salts and spirocyclic iodonium ylides. For example, incorporation of [^18^F]fluoride into the 6,10-dioxaspiro[4.5]decane-7,9-dione (SPI5) spirocyclic iodonium(III) ylide precursor of [^18^F]LY2459989 was significantly more efficient with TEAB (10 µmol, 43% RCC) as eluent than with K_2_CO_3_/K_222_ (2.5/9.3 µmol, 14% RCC) (Cai et al. 2017) The simple combination of dried [^18^F]fluoride with TEAB (10–42 µmol) and the SPI5 iodonium ylide precursor in *N,N-*dimethylformamide (DMF) has been used to prepare several clinically relevant PET radiopharmaceuticals such as [^18^F]UCB-J and [^18^F]Lorlatinib (Collier et al. 2017; Li et al. 2019b; Nkepang et al. 2016). Noteworthy, radiolabeling with dried [^18^F]TEAF generated for the first time 5-[^18^F]fluorouracil from nucleophilic [^18^F]fluoride in 11 ± 4% AY (*n* = 3) with A_m_ of 15 GBq/µmol as well as a ready to inject solution of [^18^F]FPEB in 20 ± 5% AY (*n* = 3) and with high A_m_ (666 ± 52 GBq/µmol) (Fig. 7D) (Liang et al. 2019; Rotstein et al. 2014; Stephenson et al. 2015). In addition, radiofluorination with TEAB in DMF has also been successfully applied with the spiroadamantyl-1,3-dioxane-4,6-dione (SPIAd) precursor as illustrated by the radiolabeling of [^18^F]FMT (11 ± 1% AY, *n* = 3), [^18^F]mFBG (14 ± 1% AY, *n* = 3) and 4-[^18^F]fluoro‐*m*‐hydroxy-phenethylguanidine ([^18^F]4F-MHPG, 7.8 ± 1.4% AY, *n* = 8), from which the latter two could not be directly radiolabeled using conventional nucleophilic aromatic substitution with [^18^F]KF/K_222_ (Jung et al. 2019; Rotstein et al. 2016).

### Weakly basic eluents by using polar protic solvents (R-OH)

Limited amounts of base are useful in the radiolabeling of base-sensitive precursors, but they do not always efficiently elute [^18^F]F^−^ from the anion-exchange cartridge. For example, 3.6/15 µmol K_2_CO_3_/K_222_ recovers only 18% of the radioactivity and 26 µmol TBAHCO_3_ elutes 74% of the [^18^F]F^−^ from the QMA resin (Moon et al. 2010). In order to find a proper balance between the selection of base and [^18^F]F^−^ elution efficiency, polar protic solvents have been studied extensively.

#### Potassium salts in methanol (MeOH)

**Fig. 8:**
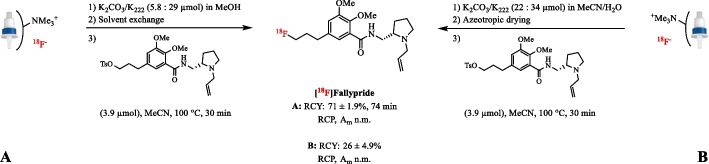
Radiosynthesis of [^18^F]Fallypride after elution of [^18^F]fluoride with a methanolic K_2_CO_3_/K_222_ solution (**A**) and the conventional K_2_CO_3_/K_222_ solution (**B**); n.m.: not mentioned

**Fig. 9:**
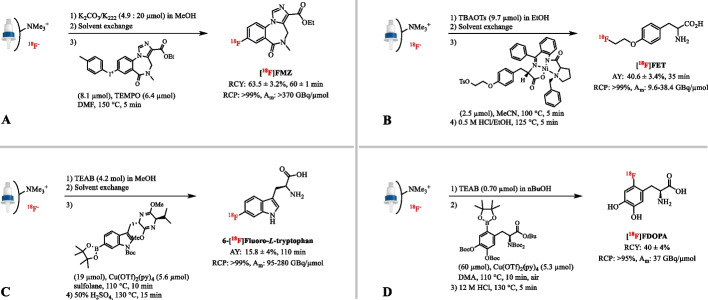
** A** Radiosynthesis of [^18^F]FMZ after elution of [^18^F]fluoride with a methanolic K_2_CO_3_/K_222_ solution. **B** Radiosynthesis of [^18^F]FET after elution of [^18^F]fluoride with the neutral phase transfer catalyst TBAOTs in ethanol. **C** Radiosynthesis of 6-[^18^F]Trp after elution of [^18^F]fluoride with alcoholic TEAB solutions. **D** Radiosynthesis of [^18^F]FDOPA after elution of [^18^F]fluoride with TEAB in nBuOH.

As shown by Moon et al., a MeOH/H_2_O (5:1) solution with 5.8 µmol K_2_CO_3_ and 29 µmol K_222_ is able to quantitatively extract [^18^F]fluoride from an anion-exchange cartridge (Moon et al. 2010). This eluent has enabled the automatic production of [^18^F]Fallypride from its base-sensitive tosylate precursor in high RCY (71 ± 1.9%, *n* = 3) compared to the classical eluent with 11/15 µmol K_2_CO_3_/K_222_ in MeCN/H_2_O (26 ± 4.9% RCY, *n* = 3) (Fig. 8). Similarly, [^18^F]FMISO could be prepared in 25.1 ± 5.0% AY (*n* = 3) using only 14 µmol of the weak base KHCO_3_ in MeOH (Lee et al. 2013). Polar protic elution systems have also been applied in Cu-mediated radiofluorination reactions of boron pinacol esters and (mesityl)diaryl iodonium salts and in the radiolabeling of diaryliodonium salts (Tang et al. 2017; Zlatopolskiy et al. 2015). In the automated preparation of [^18^F]FMZ, starting from the base labile diaryliodonium salt precursor optimal yields (80.4 ± 3.2% RCC, *n* = 3) were achieved with only 4.9 µmol K_2_CO_3_ and 15 µmol K_222_, while increasing the amount of base to 8.1 µmol K_2_CO_3_ already resulted in a significant drop in RCC (42.2 ± 4.4%, *n* = 3) (Moon et al. 2011). In order to access [^18^F]FMZ suitable for human studies, the QMA was eluted with the required low amounts of K_2_CO_3_/K_222_ in MeOH, leading to a high RCY of 63.5 ± 3.2% (*n* = 26) and high A_m_ of 370–450 GBq/µmol within 60.6 ± 1.1 min (Fig. 9A). In addition, automation of the radiosynthesis of 4-[^18^F]FGln, where the combination of KHCO_3_ and 18-Crown-6 (13/28 µmol) was proven to be important for the enantiomeric purity, resulted in superior elution efficiency (> 95%) when MeOH was used instead of MeCN/H_2_O (Qu et al. 2011; Zhang et al. 2016). This process was highly reproducible with regard to the elution efficiency and optical purity of the final product.

#### Tetrabutylammonium salts in MeOH

As with the K_2_CO_3_/K_222_ system in MeOH, limited amounts of TBAHCO_3_ (13 µmol) in MeOH are able to extract > 95% of the [^18^F]F^−^ from the QMA resin (Moon et al. 2010). This elution system also afforded [^18^F]Fallypride in a higher RCY of 71 ± 1.2% (*n* = 3) compared to the classical eluent with 39 µmol TBAHCO_3_ in MeCN/H_2_O (44 ± 4.1% RCY, *n* = 3). In addition, the formation of radiolabeled byproducts in the automated synthesis of [^18^F]FET from the Ni (II) complex of (*S*)-tyrosine Schiff base was observed in the presence of high amounts of TBAHCO_3_ (38 µmol), which was required for the quantitative elution with MeCN/H_2_O as solvent (Lakshminarayanan et al. 2017). When using a methanolic, or even ethanolic, solution of the neutral phase transfer catalyst TBAOTs (9.7 µmol), high elution efficiencies of [^18^F]F^−^ from the QMA cartridge were obtained (Fig. 9B). As a result, [^18^F]FET was prepared with A_m_ of 9.6–38.4 GBq/µmol and AY of 40.6 ± 3.4% (*n* = 5) compared to 24.6 ± 2% AY (*n* = 5) in the presence of TBAHCO_3_ (Orlovskaya et al. 2019a). In addition, very mild conditions including preconditioning of the QMA with K_3_PO_4_ (5 mmol) followed by elution with TBAOMs or TBAOTf (20 µmol) in MeOH has made the aliphatic and Cu-mediated aromatic ^18^F-labeling of highly base sensitive monosubstituted tetrazines possible (Andersen et al. 2022; Bratteby et al. 2021). As such, 2-fluoro-4-(1,2,4,5-tetrazin-3-yl)-2-[^18^F]fluoroethyl-benzoate could be obtained in 5.2 ± 2.8% RCY and with A_m_ of 96.3 ± 5.8 GBq/µmol after only 5 minutes at 100°C in *t*BuOH/MeCN (5:1). Moreover, the clinical lead compound 2,2′-(3-[^18^F]fluoro-5-(1,2,4,5-tetrazin-3-yl)benzyl)azanediyl)diacetic acid was produced in a RCY of 14 ± 3% (n = 3) and with A_m_ of 201 ± 30 GBq/µmol from the stannane precursor.

#### Tetraethylammonium salts in MeOH

TEAB (2.4–4.2 µmol) in MeOH has often been used in the Cu-mediated radiofluorination reaction of pinacol esters (Bernard-Gauthier et al. 2018; Drerup et al. 2016; Singleton et al. 2020). In the case of 6-[^18^F]fluoro-*L*-tryptophan ([^18^F]FTrp), 1 µmol TEAB resulted in a RCC of 32 ± 8%. Since such a small amount of TEAB is inappropriate for large-scale productions, methanolic TEAB (4 µmol) has been used for eluting the [^18^F]F^−^ from the cartridge during automation. Following this procedure, 6-[^18^F]Trp could be synthesized with an overall AY of 15.8 ± 4% (*n* = 4) and a A_m_ of 95–280 GBq/µmol within 110 min (Fig. 9C) (Schäfer et al. 2016; Zlatopolskiy et al. 2018). Furthermore, tetraethyl ammonium triflate (TEAOTf, 2.5 µmol) in MeOH has been successfully applied in radiofluorodestannylation reactions with the base-sensitive copper catalyst Cu(py)_4_(OTf)_2_. This eluent resulted in almost complete radioactivity recovery (95–97%) and provided several aromatic amino acids, e.g. [^18^F]FMT and [^18^F]FDOPA, in high AYs (32–54%) (Zarrad et al. 2017).

#### Tetraethylammonium salts in n-butanol (nBuOH)

To obviate an azeotropic drying or solvent-exchange step, the elution of [^18^F]F^−^ from the QMA cartridge using alcohols other than MeOH or ethanol (EtOH) have been investigated (Zischler et al. 2017). The radiolabeling of boronic and stannyl substrates often use a solution of TEAB (0.7–9.4 µmol) in *n*BuOH to elute [^18^F]F^−^ from the anion-exchange resin into a vial containing a solution of the precursor and copper catalyst in the desired solvent. Subsequently, the resulting solution is directly heated without a drying step. This general procedure has enabled the preparation of clinically relevant PET tracers including [^18^F]TRACK, [^18^F]Triacoxib, [^18^F]FDOPA and 6-[^18^F]fluorodopamine ([^18^F]FDA) (Bailey et al. 2019; Litchfield et al. 2020; Zischler et al. 2017). In the case of [^18^F]FDOPA, the unoptimized RCY (40 ± 4%, *n* = 1) was as high as the isolated RCY using the K_2_CO_3_/K_2_C_2_O_4_/K_222_ (0.72/6.0/17 µmol) eluent with an azeotropic drying step, showing the potential of this protocol (Fig. 9D) (Preshlock et al. 2016a; Zischler et al. 2017). In contrast, high A_m_ [^18^F]TRACK (188 GBq/µmol) was obtained using TEAB in *n*BuOH, but in a AY of only 4% (*n* = 1), while a AY of 8% (*n* = 1) was achieved with a solution of TEAB in MeOH (Bailey et al. 2019; Bernard-Gauthier et al. 2018). In accordance with several studies, *n*BuOH enables the most efficient ^18^F-incorporation from the alcohols investigated, however typically radioactivity recovery is around 80% resulting in a decrease in RCY (Zarrad et al. 2017; Zischler et al. 2017; Zlatopolskiy et al. 2018). The addition of more base to the eluent could increase [^18^F]F^−^ recovery to > 80%, but this can also cause a drop in RCY.

### Elution with positively charged precursor molecules in alcoholic solvents: minimalist approach

Besides potassium and tetraalkylammonium salts, labeling precursors bearing a positively charged nitrogen, iodine, sulfur or oxygen functionality (onium salts) have been investigated for the quantitative recovery of [^18^F]F^−^ from an anion-exchange resin (Feni et al. 2017; Richarz et al. 2014). Solutions of onium salts in especially MeOH can directly extract [^18^F]F^−^ from a QMA resin in excellent yields of > 95%. Low-boiling methanol can subsequently be removed completely at 70–80 °C within 2–3 min without the need for azeotropic drying. The resulting onium [^18^F]fluoride salts are directly converted into ^18^F-labeled compounds without addition of a base or any other additives under so-called minimalist conditions.

#### Onium salts in MeOH

**Fig. 10:**
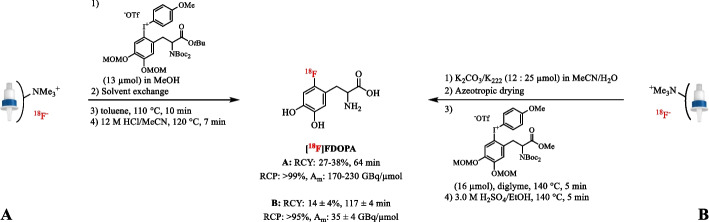
Radiosynthesis of [^18^F]FDOPA after elution of [^18^F]fluoride with the diaryliodonium precursor in an alcoholic solution (**A**) and the conventional K_2_CO_3_/K_222_ solution (**B**)

The first synthesis of 2-amino-5-[^18^F]fluoropyridines via palladium-catalyzed reaction of 2-bromo-5-[^18^F]fluoropyridine with several amines has been described using this approach (Pauton et al. 2019). The radiofluorination step of 2-bromo-5- [^18^F]fluoropyridine was performed under minimalist conditions to prevent interference of small amounts of base in the subsequent amination reaction. Especially, the application of the minimalist approach to Cu-mediated radiofluorination reactions has allowed the efficient preparation of several clinically relevant PET-tracers on a preparative scale regardless of their electronic properties, e.g. *L*-4-[^18^F]fluorophenylalanine ([^18^F]FPhe), [^18^F]DAA1106 and [^18^F]FDA (Zischler et al. 2016; Zlatopolskiy et al. 2015). The synthesis of [^18^F]FDA using the (4-anisyl)(aryl)iodonium precursor and [^18^F]KF/K_222_ in toluene was reported in 16.4 ± 3.0% AY (*n* = 4) with A_m_ >9.25 GBq/µmol in 53 ± 4 min (Vavere et al. 2014). The minimalist conditions using the same iodonium salt precursor afforded a dose of 550 MBq of [^18^F]FDA (46% RCY, *n* = 1) as a ready-to-use solution within 130 min. Similarly, Zlatopolskiy et al. reported on the (MeCN)_4_CuOTf mediated radiofluorination of the (4-anisyl)(aryl)iodonium salt precursor under minimalist conditions resulting in [^18^F]FPhe in 53–66% RCY (*n* = 3) in two steps with an A_m_ of 109 GBq/µmol compared to 20 ± 2% RCY (*n* = 2) with > 37 GBq/µmol A_m_ using the conventional method (Neumann et al. 2013; Zlatopolskiy et al. 2015). Importantly, complete removal of MeOH is a prerequisite to obtain high RCYs. Therefore, extension of the evaporation time to 5 min instead of the usual 1–2 min is advised in Cu-mediated radiofluorination reactions of aryliodonium precursors (Zischler et al. 2016). The fully automated radiofluorination of [^18^F]FDOPA from its diaryliodonium salt precursor has been improved using the minimalist approach. Under conventional conditions (12 µmol K_2_CO_3_ and 25 µmol K_222_), [^18^F]FDOPA could be produced with an A_m_ of 35 ± 4 GBq/µmol and a RCY of 14 ± 4% (*n* = 1) in 117 ± 4 min (Fig. 10) (Kuik et al. 2015). In contrast, quantitative elution of QMA-loaded [^18^F]F^−^ with the iodonium precursor (13 µmol) in MeOH (97 ± 0.03%), evaporation of MeOH and the addition of toluene yielded [^18^F]FDOPA in 27–38% RCY (*n* = 5) and high A_m_ of 170–230 GBq/µmol in 64 min (Maisonial-besset et al. 2018). As with anilinium and iodonium salts, almost complete [^18^F]F^−^ recovery from the anion-exchange cartridge has been achieved with aryl sulfonium salts in MeOH (Richarz et al. 2014; Xu et al. 2020). Importantly, the elution efficiency of onium salts is correlated with the pK_a_ value of the counteranion of the onium precursor; the stronger the basicity of the counteranion, the worse the elution of [^18^F]F^−^ (Maisonial-besset et al. 2018).

#### Onium salts in alcoholic polar aprotic solvent

**Fig. 11:**
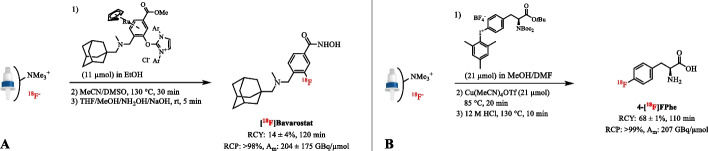
** A** Radiosynthesis of [^18^F]Bavarostat after elution of [^18^F]fluoride with the uronium precursor in an alcoholic solution. **B** Radiosynthesis of 4-[^18^F]FPhe after elution of [^18^F]fluoride with the iodonium precursor in alcoholic polar aprotic solution

The minimalist procedure has also been successfully adapted to the radiofluorination of onium salts in the absence of a solvent exchange step. In the case of uronium salts, the [^18^F]fluoride work-up procedure includes elution of [^18^F]F^−^ from the QMA with an uronium salt (11–21 µmol) in a mixture of EtOH and DMSO (Neumann et al. 2016). The elution procedure obviates the need for a solvent exchange step and subsequent heating of the resulting solution directly provides the product. However, the elution efficiency can be as low as 50% due to the low amounts of base and EtOH used. Addition of weak bases such as TBAOTf and TEAB to the eluent increased the elution efficiency, but the gain was offset by a decrease in RCY (Beyzavi et al. 2017). Under these adapted minimalist conditions, [^18^F]Bavarostat was produced under cGMP in a RCY of 14 ± 4% (*n* = 23) and an average A_m_ of 204 ± 75 GBq/µmol (Fig. 11A) (Celen et al. 2020; Strebl et al. 2017). Recently, [^18^F]Atorvastatin has been prepared using the uronium precursor in MeOH/DMSO (1/3 *v/v*), which resulted in 88 ± 5% elution efficiency, a RCY of 19 ± 6% (*n* = 10) and an A_m_ of 65 ± 32 GBq/µmol (Clemente et al. 2020). In addition, a balance between [^18^F]F^−^ recovery and ^18^F-incorporation has been achieved in the alcohol-enhanced Cu-mediated radiofluorination of (aryl)(mesityl)iodonium salts with a solution of the radiolabeling precursor (21 µmol) in 20% MeOH in DMF (Fig. 11B) (Orlovskaya et al. 2019b). A further increase of the MeOH content was detrimental and caused a rapid decrease of the ^18^F-incorporation yield due to the formation of ^18^F^–^(MeOH)_n_ clusters which substantially decrease the nucleophilicity of fluoride. Finally, ^18^F-labeled pyridines with electron‐withdrawing substituent(s) has been prepared by slow elution of [^18^F]F^–^ from the QMA cartridge with a solution of a quaternary ammonium triflate precursor in MeCN/*tert*-butanol (*t*BuOH) (1/4 *v/v*) (Basuli et al. 2018).

### Elution with base in aqueous polar aprotic solvents to form in situ OH^**−**^ ions

Wessmann et al. and describes the use of hydroxide ions (OH^−^) in anhydrous MeCN as the eluent in order to exchange with the [^18^F]F^−^ trapped on the resin (Wessmann et al. 2012). The major advantage of this method is omission of the time-consuming azeotropic evaporation of water. Although high ^18^F-incorporation yields (70–90%) were observed with fractions of the eluate of [K_222_]OH^–^, using the entire eluate resulted in only incorporation (10–20%) caused by degradation of the precursors in the presence of large amounts of OH^−^. This could be compensated by increasing the amount of precursor, but several alternative procedures have been developed using the OH^−^/[^18^F]F^−^ exchange principle. These procedures use basic ligands or other non-ionic weak bases with a high conjugate pK_a_ in the presence of trace amounts of water to generate OH^−^ in situ (Mossine et al. 2017).

#### Strong bases

**Fig. 12:**
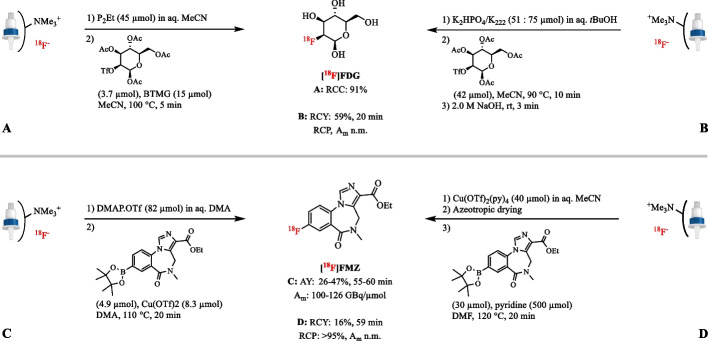
Radiosynthesis of [^18^F]FDG after elution of [^18^F]fluoride with the strong basic P_2_Et in aqueous MeCN (**A**) and the weak basic K_2_HPO_4_ in aqueous tBuOH (**b**); radiosynthesis of [^18^F]FMZ after elution of [^18^F]fluoride with the weak basic DMAP·OTf in aqueous DMF (**C**) and the metal catalyst Cu(OTf)_2_(py)_4_ in aqueous MeCN (D); n.m.: not mentioned

The first report on the in situ OH^−^ production described the strong organic base P_2_Et (45 µmol, pK_a_=32.9) in aqueous MeCN, which resulted in nearly quantitative elution of [^18^F]F^−^ (Fig. 12A) (Lemaire et al. 2010). The elution volume was smaller with eluents containing larger amounts of water or bases with higher pK_a_ values. Importantly, pK_a_ values of around 30 were required for quantitative elution of the [^18^F]F^−^ from the cartridge. It still needs to be clarified whether a base strength of this magnitude is compatible with common precursors for nucleophilic ^18^F-fluorination.

#### Weak bases

Lindner et al. used the weak basic and nucleophilic K_2_HPO_4_ in aqueous protic *t*BuOH (1% of water) as eluent in the synthesis of [^18^F]FDG (Lindner et al. 2016). The use of *t*BuOH ensured high radioactivity recovery (86 ± 5%) and enabled nearly quantitative conversion (83 ± 8%). The excellent recovery and incorporation of [^18^F]F^−^ provided [^18^F]FDG in up to 59% RCY (*n* = 1), which is comparable to the AY of 44 ± 4% (*n* = 7) obtained by Hamacher et al. using conventional fluorination conditions, but significantly faster (20 and ~ 50 min, respectively) (Fig. 12B) (Hamacher et al. 1986; Lindner et al. 2016). Finally, in the Cu-mediated radiofluorination of arylboron reagents, solutions of 4-dimethylaminopyridinium trifluoromethanesulfonate (DMAP·OTf) in aqueous DMF or *N,N-*dimethylacetamide (DMA) have been discovered as useful eluents (Mossine et al. 2017; Zhang et al. 2019b). This approach does not require azeotropic drying and the absence of base increases the stability of both precursor and the copper reagent leading to better RCYs. This approach was successfully applied in the radiosynthesis of [^18^F]FMZ (Zhang et al. 2019b). Using a solution of DMAP·OTf (82 µmol) in DMA as eluent, [^18^F]FMZ was obtained in 26–47% RCY (*n* = 3) with an A_m_ of 100–126 GBq/µmol (Fig. 12C).

#### Metal catalysts

[^18^F]Fluoride can also be eluted from the anion-exchange cartridge using an organic, aqueous solution of metal catalysts. In case of Cu-mediated radiofluorination reactions, Cu(OTf)_2_ (48 µmol), and to a lesser extent Cu(OTf)_2_(py)_4_, in DMA have been shown to be a suitable elution agent for [^18^F]F^−^ (79% elution efficiency) (Lahdenpohja et al. 2019; Mossine et al. 2017). Even though higher amounts of copper complex slightly improved the [^18^F]F^−^ recovery, large amounts of copper in the reaction solution complicated the purification of the final products on preparative scale. An example of this method has been given by Preshlock et al. in the automated production of [^18^F]FMZ with a AY of 16% (*n* = 1) using Cu(OTf)_2_(py)_4_ (40 µmol) in DMA as eluent (Fig. 12D) (Preshlock et al. 2016a). Also a solution of manganese catalysts (4–30 µmol) in pure acetone or a mixture with MeCN can directly elute [^18^F]F^−^ from an anion-exchange cartridge with > 90% efficiency (Huang et al. 2014; Liu et al. 2018). The eluate has successfully been used in the late-stage ^18^F-labeling of several unactivated aliphatic C − H bonds.

### Direct addition of [^18^F]fluoride in [^18^O]H_2_O to reaction medium with additives

An alternative to the radiofluorination on a solid-phase without a need of azeotropic drying is the use of additives in the reaction medium. These additives allow the radiolabeling reaction to take place in the presence of a significant amount of water. To this end, highly viscous solvents, metal catalysts and enzymes have been studied as additives.

#### Ionic liquids

**Fig. 13:**
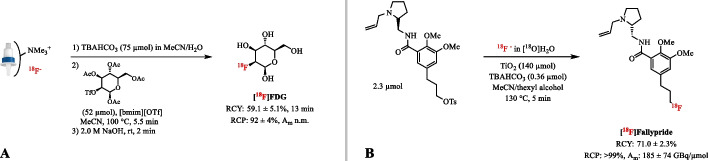
** A** Radiosynthesis of [^18^F]FDG with the ionic liquid [bmin][OTf] as additive. B Radiosynthesis of [^18^F]Fallypride with TiO_2_ as catalyst; n.m.: not mentioned

The rapid and convenient synthesis of ^18^F-fluoroalkanes has been enabled by the use of ionic liquids as reaction media, allowing the direct addition of the [^18^O]H_2_O target water containing [^18^F]fluoride and obviating the time-consuming azeotropic drying step (Kim et al. 2003, 2004). Generally, ionic liquids contain a lipophilic cation moiety based on imidazolium salts e.g. [bmim] and a counter anion that does not possess an exchangeable fluorine to prevent exchange with the [^18^F]fluoride ion. However, large amounts of starting activity are not possible, because excessive amounts of H_2_O in the reaction mixture reduce the yield of the ^18^F-fluorinated product. Besides a successful application in the synthesis of [^18^F]FDG, RCY of 59.1 ± 5.1% (dc, *n* = 3) compared to 44 ± 4% AY (*n* = 7) with the conventional method, no clinically relevant compounds or drug scaffolds have been shown (Fig. 13A) (Hamacher et al. 1986; Kim et al. 2003, 2004). A severe disadvantage of this approach is the high viscosity of these solvents, which limits their wider application.

#### Catalysts

Sergeev et al. reported the radiofluorination in up to 25 vol% of water by using titanium dioxide (TiO_2_) nanoparticles as catalyst (Sergeev et al. 2015). The TiO_2_ catalyst coordinates both aqueous [^18^F]fluoride and tosyl precursors to its surface via hydrogen bonding and thereby facilitates the reaction. In the presence of TBAHCO_3_ (0.36 µmol) as a phase- transfer agent, efficient radiolabeling of aromatic, aliphatic, and cycloaliphatic tosylated precursors has been performed without the need for a drying step. For example, the RCY and A_m_ for TiO_2_ catalyzed synthesis of [^18^F]Fallypride were found to be 71.0 ± 2.3% (*n* = 3) and 185 ± 74 GBq/µmol, respectively (Fig. 13B). This is similar to those typically obtained with conventional procedures, e.g. 66 ± 8% RCY (*n* = 8) and A_m_ of 15–78 GBq/µmol with 15 µmol TBAHCO_3_ and azeotropic drying (Lazari et al. 2014). However, increasing the amount of radioactivity in the reaction also increases the volume of water, which consequently requires a [^18^F]fluoride concentration step. In addition, triflate, nosylate and precursors with additional oxo moieties are not compatible with this method. Finally, enzymatic approaches offer a unique, mild, and selective method for the incorporation of fluorine-18 into substrates modified both on the adenine base and on the ribose ring by direct addition of [^18^F]F^−^ in [^18^O]H_2_O. The fluorinase enzyme from Streptomyces Cattleya has been successfully applied as catalyst in the ^18^F-labeling of nucleosides, e.g. [^18^F]-5′-fluoro-5′-deoxyadenosine ([^18^F]-5′-FDA), [^18^F]-5′-fluoro-5′-deoxyinosine and [^18^F]-5-fluoro-5-deoxyribose (Deng et al. 2006; Martarello et al. 2003; Winkler et al. 2008). However, the fluorinase is a relatively specific enzyme, it can never be a general fluorination reagent, and large amounts of the enzyme are required which makes purification of the product rather difficult.

### Drying of reactive [^18^F]fluoride ion from [^18^O]H_2_O

A complete methodological alternative to avoid a solvent evaporation step or the drying on a stationary phase is the conversion of aqueous [^18^F]fluoride into gaseous ^18^F-synthons or deposing it in an electric field. In both methods the radionuclide can be easily dried, either via distillation and trapping in a solvent or on an activated resin or via releasing under a low electric field. Both methods make an azeotropic drying process obsolete.

### Synthesis of reactive [^18^F]fluoride species via ^18^F-gaseous synthons – [^18^F]fluoride relay

A relatively new concept for the synthesis of reactive [^18^F]fluoride species is [^18^F]fluoride relay. In [^18^F]fluoride relay, fluorine-18 is first transferred from aqueous [^18^F]F^−^ to an easily purified ^18^F-gaseous reagent. An anhydrous [^18^F]fluoride species is subsequently released from this gas by a simple chemical or physical interaction.

#### Phosphazenium [^18^F]hydrofluoride (P_2_Et-[^18^F]HF)

**Fig. 14:**
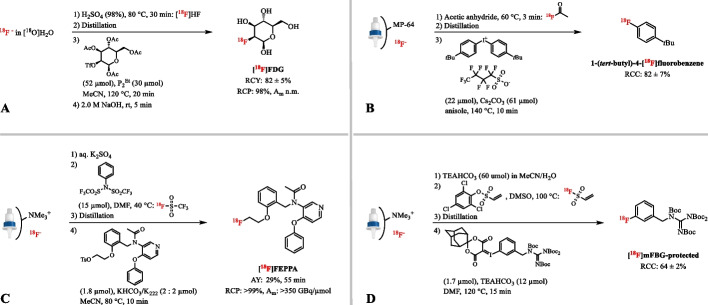
** A** Radiosynthesis of [^18^F]FDG via the gaseous [^18^F]HF synthon. **B** Radiosynthesis of 1-(*tert*-butyl)-4-[^18^F]fluorobenzene via the gaseous [^18^F]acetyl fluoride synthon. **C** Radiosynthesis of [^18^F]FEPPA via the gaseous [^18^F]triflyl fluoride synthon. **D** Radiosynthesis of protected [^18^F]mFBG via the gaseous [^18^F]ethenesulfonyl fluoride synthon; n.m.: not mentioned

Mathiessen et al. reported the use of P_2_Et-[^18^F]HF obtained by the acidification of [^18^O]H_2_O with 98% H_2_SO_4_, transfer of the formed [^18^F]HF by an argon flow and subsequent trapping on solid bound phosphazene base P_2_Et (Fig. 14A) (Mathiessen et al. 2011). Under optimal conditions, the [^18^F]HF transfer yield was 16–82%. Mannose triflate (52 µmol) was converted into [^18^F]FDG at 120 °C for 20 min without any loss of stereochemical integrity and a RCY of 82 ± 5% (*n* = 3). However, this method appears technologically demanding for automation.

#### [^18^F]Acetyl fluoride ([^18^F]AcF)

[^18^F]AcF as novel synthon for the transfer of [^18^F]fluoride through gas lines in an anhydrous form was produced by trapping [^18^F]F^−^ on an anion-exchange column (MP-64) at 60 °C followed by the reaction with acetic anhydride (Fig. 14B) (Jiang et al. 2015). The resultant gaseous [^18^F]AcF was efficiently trapped in anhydrous apolar protic solvents after purification through a column containing Porapak Q resin and sodium sulfate at 30 °C in 87 ± 3% RCY (*n* = 10) in 15 min. The purified [^18^F]AcF has also been trapped on solid-phase extraction cartridges such as Oasis WAX, but limited information is available to date. Following dissolution of [^18^F]AcF in MeCN containing TEAB (52 µmol), ^18^F-fluorination of the mannose triflate provided [^18^F]FDG in > 90% incorporation rate. Except for a few model radiofluorination reactions, the scope of [^18^F]AcF has not been investigated to date.

#### [^18^F]Triflyl fluoride

Recently the preparation of [^18^F]triflyl fluoride has been discovered (Pees et al. 2018). The reaction of N,N-bis(trifluoromethylsulfonyl)aniline with aqueous [^18^F]F^−^ yields volatile [^18^F]triflyl fluoride at 40 °C (Fig. 14C). After distillation over a sicapent column, dry [^18^F]triflyl fluoride is released in the presence of base and K_222_ in efficiencies > 90% within 5 min. The type and amount of base are variable and thus can be adapted with regard to the subsequent radiofluorination reaction. This method was used to efficiently radiolabel aromatic and aliphatic compounds in moderated to high RCYs as well as the PET tracers [^18^F]FES and [^18^F]FET in good RCYs (17–57% and 10–55%, respectively, *n* = 2) and A_m_ of up to 123 GBq/µmol. A comparable range in molar activity and RCYs was observed using the standard elution and drying procedure (Bourdier et al. 2011a; Fedorova et al. 2018). As an extension of this method, Dahl et al. showed the in-loop radiofluorination with [^18^F]triflyl fluoride as the labeling agent (Dahl et al. 2019). *N*-acetyl-*N*-(2-[^18^F]fluoroethoxybenzyl)-2-phenoxy-5-pyridinamine ([^18^F]FEPPA) and 7-(6-[^18^F]fluoropyridin-3-yl)-5 H-pyrido[4,3-β]indole ([^18^F]T807) were synthesized with AYs of 29% (*n* = 1) and 27% (*n* = 1), respectively, and A_m_ >350 GBq/µmol for both radiotracers. The in‐loop radiofluorination methodology provided equal or superior yields compared with conventional reactions, e.g. the syntheses of [^18^F]T807 and [^18^F]FEPPA have previously been reported in AYs of 14 ± 3% (*n* = 3) and 38 ± 3% (*n* = 15), respectively (Berroteran-Infante et al. 2018; Shoup et al. 2013).

#### [^18^F]ethenesulfonyl fluoride ([^18^F]ESF)

[^18^F]ESF forms a simple way of activating [^18^F]fluoride and can conveniently be shipped for off-site use (Zhang et al. 2019a). [^18^F]ESF is produced from [^18^F]fluoride and 2,4,6-trichlorophenylethenesulfonate in the presence of TEAB (60 µmol) and subsequently distilled onto a silica-plus cartridge with an average RCY of 57 ± 11% (Fig. 14D). [^18^F]ESF was converted into a reactive [^18^F]fluoride species by elution with TEAB (12 µmol) in a chosen solvent. The RCCs using [^18^F]ESF method were comparable to the use of traditionally dried [^18^F]TEAF complex in the case of clinically used aliphatic and aromatic compounds. An exception were boron and stannane precursors in Cu-mediated ^18^F-labeling reactions where the RCYs obtained using [^18^F]ESF were lower than the ones obtained using [^18^F]TEAF. This might be due to the use of [^18^F]TEAF instead of [^18^F]KF/K_222_ complexes reported previously, to different automated drying sequences or to other currently uncontrolled parameters. It was also observed that the standard deviations for the radiofluorinations were generally lower when using [^18^F]ESF, which could be linked to the consistent quality of this labeling reagent compared to the [^18^F]TEAF coming from a drying process that might introduce varying grades of moisture.

### Synthesis of anhydrous [^18^F]fluoride via electrochemical deposition and magnetic droplets

#### Electrochemical deposition

Instead of anion-exchange resins, concentration of [^18^F]fluoride from the target water has also been performed using electrodeposition (Cardinale et al. 2014; Hamacher et al. 2002; Hamacher and Coenen 2006; Kronenberg et al. 2007; Melean et al. 2011; Meleán et al. 2015; Reischl et al. 2002; Sadeghi et al. 2013; Saito et al. 2007; Wagner et al. 2009). Basically, [^18^F]fluoride from the water target is deposited on the anodic site of an electrochemical cell after which it is recovered under reversed voltage in aprotic solvent containing a phase transfer catalyst. Although this method has proven to be not very efficient, its convenience has led to several applications, especially in microfluidic devices. For example, Saiki et al. reported a microfluidic cell that could trap [^18^F]fluoride from 1 to 2 mL of [^18^O]H_2_O by applying a potential and then release the activity into 275 µL of eluent with an overall efficiency of ~ 60% (Saiki et al. 2010; Wong et al. 2012). An alternative platform makes use electrowetting-on-dielectric (EWOD) (Chen et al. 2014; Keng et al. 2012). An EWOD microfluidic chip typically consists of two parallel plates, one patterned with electrodes and one coated with a conductor to act as a ground electrode. An electrical potential is then applied to the desired electrodes to achieve operations such as droplet transport or heating. For example, a 200 µL droplet of [^18^F]fluoride in [^18^O]H_2_O with base and phase transfer catalyst is loaded and its volume reduced by heating the nearby electrodes. An electrical potential is then applied to the electrodes to move the droplet to a heating site. Here, the target water is further removed by evaporation resulting in a residue of 5 µL in 10 min. [^18^F]FLT and [^18^F]Fallypride have been successfully synthesized on an EWOD device in 63 ± 5% RCY (*n* = 5) and 65 ± 6% RCY (*n* = 7), respectively (Javed et al. 2014a, b). These yields are comparable to, or greater than, those obtained by conventional approaches e.g. 40% RCY (*n* = 1) for [^18^F]FLT and 66 ± 8% RCY (*n* = 8) for [^18^F]Fallypride (Lazari et al. 2014; Suehiro et al. 2007). In addition, the A_m_ of [^18^F]Fallypride (730 GBq/µmol) was two times higher on the EWOD device than synthesized in a macroscale radiosynthesizer (330 GBq/µmol), despite starting with significantly less radioactivity (270 MBq and 7400 MBq, respectively). Although this approach is suitable for modest starting volumes, impractically large chips and many time-consuming sequential 200 µL evaporations are required to handle volumes of ~ 2 mL of [^18^F]fluoride in enriched water that are generated in most cyclotrons.

#### Magnetic droplet microfluidics (MDM)

Finally, Fiel et al. described the use of magnetic particles to manipulate [^18^O]H_2_O droplets in the [^18^F]fluoride preconcentration step (Fiel et al. 2015). A magnet is moved to manipulate the magnetic particles which hold the small target water droplet by surface tension, but also possess an anion-exchange property that is capable of capturing and releasing the [^18^F]F^−^. The capture efficiency was 79% using low initial activities (~ 74 MBq) and 25 mg of magnetic particles. After evaporation of the solvent, [^18^F]F^−^ is released with 50 µL of K_2_CO_3_ (7.3 µmol) in 91–95% efficiency. The preconcentration step took approximately 5 min and the [^18^F]fluoride solution was preconcentrated by 15-fold. However, it was found that when the initial ^18^F-activity was increased to ~ 300 MBq, the capture efficiency already decreased to ca. 59%, which makes clinical application questionable.

## Conclusions

The large number of publications on the preparation of reactive [^18^F]fluoride from oxygen-18 enriched water over the last decade reflects the growing importance of the complex activation step in ^18^F-fluorination chemistry. Success and progress have been achieved in base-sensitive nucleophilic ^18^F-fluorination reactions, providing improved, modified or even completely new fluorine-18 work-up procedures. The currently most used elution system still comprises the conventional K_2_CO_3_/K_222_ eluent, but the mild base tetraethylammonium bicarbonate (TEAB) as phase-transfer catalysts has been shown to be a useful alternative in the ^18^F-labeling of iodonium(III) containing precursors, e.g. [^18^F]UCB-J. In addition, the moderately basic K_2_CO_3_/K_2_C_2_O_4_/K_222_ eluent as well as positively charged precursors in alcoholic medium, i.e. the minimalist approach, in especially Cu-mediated nucleophilic ^18^F-fluorination reactions have found widespread use.

It should be mentioned that many developments listed in this review only comprise small adaptations compared to the conventional methodology and are focused on a specific reaction or substrate. Hence, several of these methods have already proven their advantage and suitability for ^18^F-labeled tracer synthesis, but more comparative evaluations are desirable. In contrast, new approaches have been reported and hold promise, but often only concern specific reactions with primarily simple substrates as application. From these methods, the recent [^18^F]triflyl fluoride procedure has to be mentioned as a successful example of a new [^18^F]fluoride work-up methodology because of its use in clinical PET tracer production.

Despite the large variety of new activation procedures, none has completely replaced the conventional K_2_CO_3_/K_222_ elution method and further expansion of miniaturization and kit-like procedures is an area of research with room for improvement. Automated cassettes and technologies like microfluidic devices to produce reactive [^18^F]fluoride will increasingly be used in PET chemistry by providing safe, standardized and automated GMP production tools. Considering the many promising developments going on, there is great confidence in further inventions and the establishment of a few standardized drying methodologies for the routine production of a broad scale of PET tracers.

## Data Availability

Not applicable.

## Abbreviations

[^18^F]-5′-FDA: [^18^F]-5′-fluoro-5′-deoxyadenosine
[^18^F]-L-FPHCys: *L-S*-(3-[^18^F]fluoropropyl)homocysteine
[^18^F]4 F-MHPG: 4-[^18^F]fluoro‐*m*‐hydroxy-phenethylguanidine
[^18^F]AcF: [^18^F]acetyl fluoride
[^18^F]DAA1106: *N*-(2,5-dimethoxybenzyl)-*N*-(5-[^18^F]fluoro-2- phenoxyphenyl)acetamide
[^18^F]DCFPyL: 2-(3‐{1‐Carboxy‐5‐[(6‐[^18^F]fluoro‐pyridine‐3‐carbonyl)‐amino]‐pentyl}‐ureido)‐pentanedioic acid
[^18^F]ESF: [^18^F]ethenesulfonyl fluoride
[^18^F]Fallypride: (*S*)-*N*-[(1-allyl-2-pyrrolidinyl)methyl]-5-(3-[^18^F]fluoropropyl)-2,3-dimethoxybenzamide
[^18^F]FAZA: [^18^F]fluoroazomycin arabinosid
[^18^F]FDG: 2-[^18^F]fluoro-2-deoxy-*D*-glucose
[^18^F]FDHT: 16β-[^18^F]fluoro-5α-dihydrotestosteron
[^18^F]FDOPA: 6-[^18^F]fluoro-*L*-dihydroxy-phenylalanine
[^18^F]FDPA: *N,N*-diethyl-2-(2-(4-[^18^F]fluorophenyl)-5,7-dimethylpyrazolo[1,5- a]pyrimidine-3-yl)-acetamide
[^18^F]FEPPA: *N*-acetyl-*N*-(2-[^18^F]fluoroethoxybenzyl)-2-phenoxy-5-pyridinamine
[^18^F]FES: 16α-[^18^F]fluoroestradiol
[^18^F]FET: *O*-(2-[^18^F]fluoroethyl)-*L*-tyrosine
[^18^F]FLT: 3′-Deoxy-3′-[^18^F]fluorothymidine
[^18^F]FMAU: 2′-Deoxy-2′-[^18^F]fluoro-5-methyl-1-β-*D*-arabinofuranosyluracil
[^18^F]FMISO: 3-[^18^F]fluoro-1-(2′-nitro-1′-imidazolyl)-2-propanol
[^18^F]FMT: *L*-3-[^18^F]fluoro-α-methyltyrosine
[^18^F]FMZ: [^18^F]Flumazenil
[^18^F]FP-CIT: *N*-[^18^F]fluoropropylcarbomethoxyiodophenyl-nortropane
[^18^F]FPEB: [^18^F]-3-fluoro-5-[(pyridin-3-yl)ethynyl]benzonitrile
[^18^F]FPhe: *L*-4-[^18^F]fluorophenylalanine
[^18^F]FTrp: 6-[^18^F]fluoro-*L*-tryptophan
[^18^F]mFBG: *meta*-[^18^F]fluorobenzylguanidine
[^18^F]T807: 7-(6-[^18^F]fluoropyridin-3-yl)-5 H-pyrido[4,3-β]indole
[^18^O]H_2_O: Oxygen-18 enriched water
3-[^18^F]F-NNDI: *N*-(3-fluoro-4-nitronaphthyl)-*cis*-5-norbornene-endo-2,3-dicarboxylic imide
4-[^18^F]FGln: (2* S*,4*R*)-4-[^18^F]fluoroglutamine
A_m_: Molar activity
AY: Activity yield
DMA: *N,N*-dimethylacetamide
DMAP·OTf: 4-Dimethylaminopyridinium trifluoromethanesulfonate
DMF: *N,N*-dimethylformamide
EtOH: Ethanol
EWOD: Electrowetting-on-dielectric
GMP: Good manufacturing practice
K_222_: kryptofix-222
MeCN: Acetonitrile
MeOH: Methanol
*n*BuOH: *n*-butanol
P_2_Et-[^18^F]HF: Phosphazenium [^18^F]hydrofluoride
PET: Positron emission tomography
RCC: Radiochemical conversion
RCY: Radiochemical yield
SCIDY: Spirocyclic iodonium ylides
SPI5: 6,10-Dioxaspiro[4.5]decane-7,9-dione
SPIAd: Spiroadamantyl-1,3-dioxane-4,6-dione
TBACN: Tetrabutylammonium cyanide
TBAHCO_3_: Tetrabutylammonium bicarbonate
TBAOH: Tetrabutylammonium hydroxide
TBAOMs: Tetrabutylammonium methanesulfonate
TBAOTf: Tetrabutylammonium triflate
*t*BuOH: *tert*-butanol
TEAB: Tetraethylammonium bicarbonate
TEAOTf: Tetraethyl ammonium triflate
